# Dacryocystitis presenting as post-septal cellulitis: a case report

**DOI:** 10.1186/1752-1947-1-77

**Published:** 2007-09-05

**Authors:** Scott E Henney, Mike J Brookes, Kevin Clifford, Anirvan Banerjee

**Affiliations:** 1 ENT Department, James Cook University Hospital, Marton Road, Middlesbrough, UK; 2 Radiology Department, James Cook University Hospital, Marton Road, Middlesbrough, UK; 3 50 Lyndon Road, Sutton Coldfield, West Midlands, WS9 0RJ. UK

## Abstract

Dacryocystitis is relatively common, the majority of patients present with pre-septal cellulitis and not an orbital abscess due to anatomical barriers. The authors report a case of dacryocystitis presenting as post-septal cellulitis in a postmenopausal lady with an underlying malignancy. Following antibiotic therapy and elective dacryocystorhinostomy the patient is still under follow-up, and has no further recurrence of symptoms. Orbital abscess in postmenopausal women presenting with dacryocystitis should be considered, as prompt recognition and early surgical intervention is required to prevent visual loss.

## Background

Dacryocystitis is associated with pyrexia and severe erythematous swelling around the nasal aspect of the lower lid. The majority of patients with dacryocystitis present with pre-septal cellulitis and not an orbital abscess. Orbital abscess formation and can lead to vision loss therefore requires emergency surgical drainage.

## Case Presentation

A 50-year-old woman was referred to the Ear Nose and Throat department with a two day history of a painful swollen right eye. She was an in-patient awaiting wide local excision and axillary node clearance with post-operative chemotherapy for receptor-negative carcinoma of the breast.

Examination revealed marked swelling and erythema of both upper and lower lids of the right eye. Swelling prevented complete visualisation of the pupil and cornea. There was mild proptosis and a restriction of extraocular motility. Visual acuity was measured at 6/60 on the left and 4/60 on the right. Examination of the other cranial nerves, nose, nasopharynx and neck was normal.

**Figure 1:**
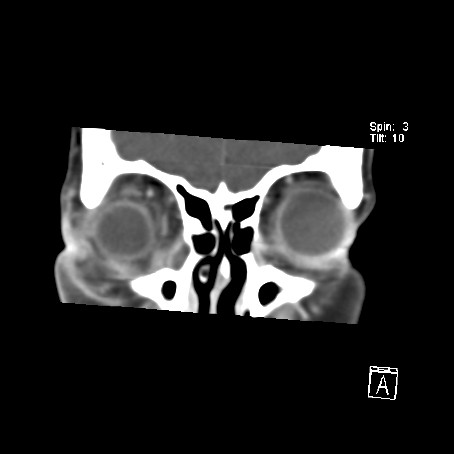
CT scan of the orbits and brain, coronal view. Soft tissue density collection on the floor of the right orbit, elevating the inferior rectus muscle, extending posteriorly to the apex of the orbit.

**Figure 2:**
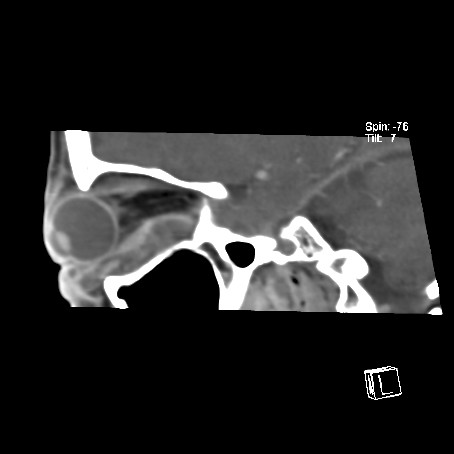
CT scan of the orbits and brain, sagittal view. There appeared to be a more focal peripherally enhancing collection just inferior to the globe and a further enhancing collection adjacent to and within the fossa for the lacrimal sac.

**Figure 3:**
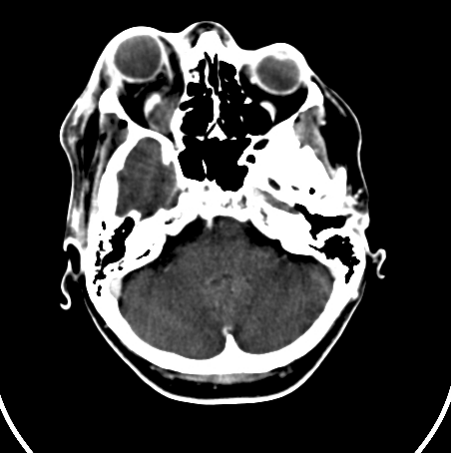
CT scan of the orbits and brain, axial view. Streaky increased density was noted within the intra-coronal and extra-coronal fat, and there was right proptosis.

**Figure 4:**
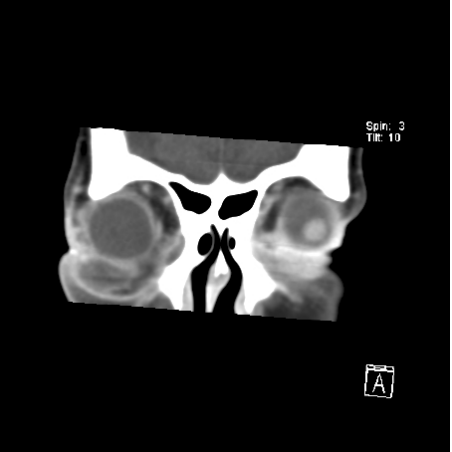
CT scan of the orbits and brain, coronal view. The swelling extended into the soft tissues of the infra-orbital region, but there was little pre-septal soft tissue swelling.

A computed tomography (CT) scan of the orbits and brain demonstrated a soft tissue density collection on the floor of the right orbit, elevating the inferior rectus muscle, extending posteriorly to the apex of the orbit (Fig 1). There appeared to be a more focal peripherally enhancing collection just inferior to the globe and a further enhancing collection adjacent to and within the fossa for the lacrimal sac (Fig 2). Streaky increased density was noted within the intra-coronal and extra-coronal fat, and there was right proptosis (Fig 3). The swelling extended into the soft tissues of the infra-orbital region, but there was little pre-septal soft tissue swelling (Fig 4). The para-nasal sinuses and nasal cavity appeared normal, as did the canal for the nasolacrimal duct.

The features identified on the CT scan were consistent with an inflammatory process and the enhancing rim around the fossa for the lacrimal sac was consistent with dacryocystitis.

The patient underwent drainage of orbital abscess via an orbital rim incision and the purulent material was sent for microbiological investigation. Intraoperatively, dilation of nasolacrimal sac was noted. The diagnosis of dacryocystitis with tracking posteriorly was confirmed. The purulent material cultured demonstrated a mixed growth of coliforms, sensitive to ciprofloxacin. The patient responded well to intravenous ciprofloxacin and metronidazole. She regained full extraocular motility, the proptosis resolved and her vision was 6/6 on the left and 6/12 on the right at discharge.

One month following surgery the patient presented with a red, swollen, tender right eye. A diagnosis of early recurrence of dacryocystitis was made. CT orbits was repeated, and no compressive lesion to account for her nasolacrimal duct problem was shown. This episode settled with ciprofloxacin and metronidazole. She was found to have a right nasolacrimal duct stenosis and underwent right endonasal dacryocystorhinostomy (DCR) to prevent further abscess formation. The patient is still under follow-up, and has no further recurrence of symptoms.

## Discussion

Dacryocystitis is associated with pyrexia and severe erythematous swelling around the nasal aspect of the lower lid [7]. The majority of patients with dacryocystitis present with pre-septal cellulitis and not an orbital abscess. The reason for this seems to be the insertion of the orbital septum on the posterior orbital crest preventing extension to the orbit [4]. Other anatomical barriers exist, including lacrimal fascia, medial canthal ligament and obicularis muscle [6]. Once these barriers have been breached orbital abscess formation is unimpeded and can lead to vision loss, requiring surgical drainage. Post-septal cellulitis is more commonly associated with congenital dacryocystitis as the orbital septum is poorly formed in infants. There are only a few documented cases of post-septal spread in adults [1-6].

Dacryocystitis is relatively common in the general population, with the majority of cases seen in the first and fifth decade of life, especially in postmenopausal women (70–83% of cases) and those with poor hygiene [7]. The prevalence of dacryocystitis in the postmenopausal population may be due to changes in the size of the nasolacrimal duct anatomy. Groessl et al demonstrated that women have significantly smaller dimensions in the lower nasolacrimal fossa and middle nasolacrimal duct [9]. Moreover, there were changes noted in the antero-posterior dimensions of the bony nasolacrimal canal coinciding with the osteoporotic disease process occurring in middle-aged females [9]. Hormonal changes, which produce a generalised de-epithelialisation, may cause a de-epithelialisation in the lacrimal sac and duct, resulting in an already narrowed canal becoming blocked [7].

Underlying malignancy may also impair inflammatory and immunological responses to infection. Obstruction of the nasolacrimal duct due to metastatic spread has been reported with primary sites from the breast and prostate, but is an extremely rare phenomenon and there was no evidence that this occurred in this case [10].

## Conclusion

Orbital complications of dacryocystitis are rare because of the septum and other anatomical barriers. In postmenopausal women, the osteoporotic process causes changes in the dimensions of the bony nasolacrimal canal, and hormonal changes may cause desquamation and consequently blocking of the canal. The possibility of an orbital abscess in postmenopausal women presenting with dacryocystitis should be considered, as prompt recognition and early surgical intervention is required to prevent visual loss. Elective dacryocystorhinostomy once the acute infectious phase has settled is the treatment of choice in adult patients [4,8].

## Abbreviations

Dacryocystorhinostomy (DCR)

Computed tomography (CT)

## Competing interests

The author(s) declare that they have no competing interests.

## Authors' contributions

S.E. Henney participated in the case report format, performed literature review and drafted the manuscript.

M.J. Brookes participated in literature review and helped to draft the manuscript.

A. Banerjee conceived of the case report, and participated in its design and coordination and helped to draft the manuscript.

K Clifford performed and interpreted the images and helped to draft the manuscript.

All authors read and approved the final manuscript.
